# Surgical ambulance referrals in sub-Saharan Africa – financial costs and coping strategies at district hospitals in Tanzania, Malawi and Zambia

**DOI:** 10.1186/s12913-021-06709-5

**Published:** 2021-07-23

**Authors:** Martilord Ifeanyichi, Henk Broekhuizen, Mweene Cheelo, Adinan Juma, Gerald Mwapasa, Eric Borgstein, John Kachimba, Jakub Gajewski, Ruairi Brugha, Chiara Pittalis, Leon Bijlmakers

**Affiliations:** 1 Department for Health Evidence, Radboud Institute for Health Sciences, Radboud University Medical Centre, Nijmegen, The Netherlands; 2 EMAI Health Systems and Health Services Consulting, Nijmegen, The Netherlands; 3 Surgical Society of Zambia, Department of Surgery, University Teaching Hospital, Lusaka, Zambia; 4 East, Central and Southern Africa Health Community, Arusha, Tanzania; 5 College of Medicine, University of Malawi, Blantyre, Malawi; 6 Institute of Global Surgery, Royal College of Surgeons in Ireland, Dublin, Ireland; 7 Department of Public Health and Epidemiology, Royal College of Surgeons in Ireland, Dublin, Ireland

**Keywords:** Global surgery, Referral systems, Costs, Sub-Saharan Africa, District hospital, Ambulance

## Abstract

**Background:**

An estimated nine out of ten persons in sub-Saharan Africa (SSA) are unable to access timely, safe and affordable surgery. District hospitals (DHs) which are strategically located to provide basic (non-specialist) surgical care for rural populations have in many instances been compromised by resource inadequacies, resulting in unduly frequent patient referrals to specialist hospitals. This study aimed to quantify the financial burdens of surgical ambulance referrals on DHs and explore the coping strategies employed by these facilities in navigating the challenges.

**Methods:**

We employed a multi-methods descriptive case study approach, across a total of 14 purposively selected DHs; seven, three, and four in Tanzania, Malawi and Zambia, respectively. Three recurrent cost elements were identified: fuel, ambulance maintenance and staff allowances. Qualitative data related to coping mechanisms were obtained through in-depth interviews of hospital managers while quantitative data related to costs of surgical referrals were obtained from existing records (such as referral registers, ward registers, annual financial reports, and other administrative records) and expert estimates. Interview notes were analysed by manual thematic coding while referral statistics and finance data were processed and analysed using Microsoft Office Excel 2016.

**Results:**

At all but one of the hospitals, respondents reported inadequacies in numbers and functional states of the ambulances: four centres indicated employing non-ambulance vehicles to convey patients occassionally. No statistically significant correlation was found between referral trip distances and total annual numbers of referral trips, but hospital managers reported considering costs in referral practices. For instance, ten of the study hospitals reported combining patients to minimize trip frequencies. The total cost of ambulance use for patient transportation ranged from I$2 k to I$58 k per year. Between 34% and 79% of all patient referrals were surgical, with total costs ranging from I$1 k to I$32 k per year.

**Conclusion:**

Cost considerations strongly influence referral decisions and practices, indicating a need for increases in budgetary allocations for referral services. High volumes of potentially avoidable surgical referrals provide an economic case – besides equitable access to healthcare – for scaling up surgery capacity at the district level as savings from decreased referrals could be reinvested in referral systems strengthening.

**Supplementary Information:**

The online version contains supplementary material available at 10.1186/s12913-021-06709-5.

## Introduction

Despite growing global attention to surgery [1, 2], its recognition as a crucial component of health rights [3], and growing evidence of its cost-effectiveness [4–6], surgical care in sub-Saharan Africa (SSA) falls short of what people need. An estimated nine out of ten persons in this region are unable to access timely, safe and affordable surgery [7]. This results in poor health outcomes [8] and substantial economic losses [5]. A key strategy for meeting the unmet need for surgery lies in the strengthening of district health systems [9, 10]. Most health systems in SSA have a pyramidal structure, with numerous community providers (health centres, dispensaries, health posts, and sometimes outreach clinics offered by mobile teams) at the base. These refer patients to district hospitals (DHs) when needed. DHs in turn refer complex cases to a limited number of regional/provincial/zonal hospitals, and only cases that require more specialized care are referred onwards to and are handled by national/central hospitals that constitute the apex of the pyramid.

By design, DHs occupy a critical position in the national healthcare pyramid. They provide emergency surgical care to rural populations; serve as first referral points, providing services to patients who would otherwise face financial challenges accessing services at higher-level referral hospitals (RHs) in major cities [11]; and they function as gate-keepers for the latter. They therefore ensure that only complex cases are managed in the high cost environment of specialist hospitals. In reality however, DH functions in SSA have in most cases been compromised by systemic inefficiencies such as inadequate funding [9, 10, 12], poor infrastructure, shortages of supplies, and deficiencies in manpower [13, 14], resulting in a general loss of confidence in district-level health services. Patients often go straight to higher-level hospitals, bypassing the DHs close to them. This leads to essential non-complex surgery congesting referral hospitals [15]. Even healthcare workers gravitate to the RHs. A vicious cycle is effectively established as poorly utilized DHs lose the required political momentum for funding thereby further undermining them [16]. Consequently, DHs refer inappropriately high volumes of cases – electives and emergencies – to the higher centres [15, 17].

Studies on ambulance transport in SSA have focused mainly on emergency obstetric care for rural women, especially since the turn of the millennium when such care was identified as crucial to the achievement of MDGs 4 and 5 [18–21]. These studies nevertheless provide useful insights into the dynamics of ambulance services within health systems generally. For instance, studies of the effectiveness of ambulance referrals have shown positive health outcomes [18, 22, 23]; economic studies have reported ambulance referrals to be cost-effective [24–26] and cost-efficient [23, 27]. Despite these, concerns remain about the high costs of purchase and operations of ambulances [20], including poor maintenance and frequent breakdowns [20, 25]. These have informed trials using low-cost alternatives such as bicycles in Malawi [28]; motorcycles in Zambia [20], Sierra Leone [29], and Malawi [25]; and contracts with private transport service providers in Nigeria [30].

The literature on surgical referrals in SSA is scanty, and the few available studies have concentrated on investigating referral patterns [15, 17, 31, 32] and to a lesser extent quality of referrals [17, 33]. All were conducted at the level of tertiary hospitals, ignoring the perspective of the referring facilities which are often DHs. The operations and economic dimensions of transport systems have been under-researched [34]. Surgical referrals, according to multiple authors, place heavy financial loads on already financially constrained DHs [12, 13, 35]; but to the best of our knowledge these have never been quantified. In addition, no study has looked at coping strategies deployed to limit the costs of patient transportation by ambulance at DHs in SSA. An understanding of the (economic) dynamics of and improvements in ambulance referrals at the first level hospitals would contribute to more effective and efficient surgery delivery across all the levels of care [33].

Two questions guided the study reported here: First, what are the financial burdens on DHs of ambulance referrals generally and surgical ambulance referrals specifically? Second, how do hospitals manage the economic challenges associated with ambulance referral services in a resource-constrained environment?

## Methods

This study aimed to describe the practices and quantify the financial burdens of ambulance referrals of surgical patients from the perspective of the DHs. Based on published literature [25] and the authors’ knowledge of the country settings, three cost elements were identified: fuel consumption, vehicle maintenance and staff allowances. We employed a multi methods, multi-centre descriptive case study approach.

### Study setting

This study was conducted as part of the economic evaluation and policy analyses of the Scaling Up Safe Surgical Services for Rural and District Populations in Africa (SURG-Africa) project. SURG-Africa is an EU-funded four-year implementation research project that aims to scale up safe accessible surgery in rural parts of Tanzania, Malawi and Zambia through a surgical supervision model, enabling surgeon specialists to be trainers and mentors of district hospital staff [36]. The socioeconomic and surgical indices of the three countries are presented in Table 1.

**Table 1:** Selected Socioeconomic and Surgical Indices of Tanzania, Malawi and Zambia

	Tanzania	Malawi	Zambia
^a^National population	53 M	17 M	16 M
^a^GDP (current US$)	50B	5B	21B
^a^GDP per capita (current US$)	967	316	1, 281
^a^Current health expenditure (% of GDP) health	4.1	9.8	4.5
^a^Domestic government general health expenditure (% of total govt expenditure)	9.5	9.8	7.1
^a^SOA specialists (per 100,000 population)	0.5	0.4	1.5
Surgery volumes (per 100,000 population, per year)	484^b^	747^d^	1600^f^
Populations without (geospatial) access to surgical care (%)	N/A	N/A	81^g^
% of annual mortality due to surgical conditions	19^c^	24^e^	N/A
Surgical burden of disease (% of the population)	N/A	35^e^	N/A
^a^Proportion of the population at risk of catastrophic health expenditure (%)	49	90	N/A
^a^Proportion of the population at risk of impoverishing health expenditure (%)	68	100	N/A

^a^World Bank Development Indicators, 2016; ^b^Citron et al., 2019 (DOI: 10.1136/bmjgh-2018-001282); ^c^Institute for Health Metrics and Evaluation, 2016; ^d^Henry et al., 2014 (DOI: 10.1093/heapol/czu102); ^e^Varela et al., 2017 (DOI: 10.4314/mmj.v29i3.1); ^f^Weiser et al., 2016 (DOI: 10.2471/BLT.15.159293); ^g^Esquivel et al., 2016 (DOI: 10.1001/jamasurg.2016.2303); N/A = Not Available; GDP = Gross Domestic Product; SOA = Surgical, Obstetric and Anaesthesia

There are differences in the health system structures and staffing patterns of the countries. Tanzania operates a four-level pyramidal health structure: primary level facilities (including the DHs), regional hospitals, zonal hospitals, and national hospitals. Surgeries at district hospitals are performed by medical officers - MOs (post-internship medical doctors) and assistant medical officers (AMOs). AMOs are non-physician clinicians with advanced diploma training in medicine and surgery.

The Malawi health system has a three-level structure comprising: (1) primary level facilities (health centres and dispensaries); (2) secondary level facilities (DHs and mission hospitals [MHs] of equivalent capacity); (3) tertiary facilities (the central/specialized/specialist hospitals). Surgeries at DHs are mainly performed by clinical officers (COs), and to a lesser extent by MOs and “clinical associates”. COs are non-physician clinicians with a (three year) diploma training in medicine and surgery, while clinical associates are COs with a bachelor’s degree in surgery (or paediatrics or obstetrics and gynaecology).

A three level system of care in Zambia comprises: (1) first level facilities with the district hospital at the apex; (2) second level facilities (provincial or general hospitals); and (3) third level facilities (specialist/teaching/central hospitals). District surgeries are performed here by MOs and medical licentiates (MLs), who are non-physician clinicians with an advanced diploma or bachelor’s degree training, including surgery and anesthesia as major components.

None of the three countries have national referral guidelines, despite national health policies emphasizing the importance of strong referral systems [37–39].

### Site selection

A total of 14 DHs were selected purposively, among intervention facilities of the SURG-Africa project. In Tanzania, seven out of twelve DHs were selected in Arusha and Kilimanjaro regions, based on higher volumes of surgery output, while ensuring inclusion of both government and faith-based facilities. In Zambia, one and three facilities were chosen in Central and Southern provinces respectively, with the aim of representing the variations in distance to the tertiary hospitals and hospital ownership (government and church). Three facilities were selected in Malawi’s Southern region, because of their prior relationships with the researchers.

### Data collection and sources

Data collection was conducted by author MI in June 2019 in Tanzania; authors MI and LB from June to July 2019 in Malawi; and authors MI and MC in November 2019 in Zambia. Tanzania and Malawi data pertain to the July 2018/June 2019 financial year while the Zambian data reflect the 2018 calendar year. Exceptions to these time horizons were Oltrumet and Meru DHs in Tanzania where 2017/2018 data were used instead, and Nangoma MH in Zambia where May 2017 – June 2018 data were used. Qualitative data related to coping mechanisms were obtained through in-depth interviews while quantitative data related to costs of surgical referrals were obtained from records/documents. An iterative combination of the two enabled cross-augmentation and triangulation.

We conducted in-depth interviews with district health/hospital staff members. Respondents were broadly targeted based on their involvement in patient care and referral decisions, finance and administration, and/or ambulance movements. A total of 60 respondents were interviewed: 28, 17 and 15 in Tanzania, Malawi and Zambia, respectively (presented in Additional file 1). Semi-structured interviews were administered based on a pre-designed interview guide (presented in Additional file 2), with sub-topics around referral infrastructure, referral centres and distances, referral frequencies, use of ‘escort personnel’, use of referral registers, purposes of ambulance use, payment of allowances, diesel procurement and management, patient combination in trips, and administrative practices. Interviews lasted 45–60 min and were conducted mostly one-on-one, at the respondents’ work stations. Interview responses were typed verbatim into a laptop. Tape recording was cancelled after initial attempts were received with reservations.

Numbers of ambulance trips were obtained mainly from logbooks and administrative records. Actual lists of cases referred were obtained from either the referral registers (where available) or ward registers. Since, there were no dedicated surgical referral registers or dedicated surgical wards, we identified (together with local clinicians, where necessary) which cases were surgical, based on the recorded diagnoses. Table 2 presents the overview of the quantitative data and sources.

**Table 2:** Quantitative Data Sources

Data Element	Source
Numbers (lists) of cases referred	Referral registers, ward registers
Numbers of ambulance trips	Vehicle log books, administrative records, referral registers, interviews (estimates)
Distances covered	Vehicle log books, Google Maps
Fuel consumption	Fuel register, fuel ledger, interviews (estimates), annual financial reports
Vehicle maintenance costs and taxes	Annual financial reports, administrative records
Costs of allowances	Annual financial reports, administrative records

### Delineation

A “surgical referral” was defined as any referral from a DH that was reasonably expected to be managed by a surgeon or an obstetrician/gynaecologist, and could potentially be treated operatively at the RH, irrespective of the final treatment approach. Surgical cases referred primarily because of medical comorbidities (e.g. uncontrolled hypertension or diabetes mellitus) were considered as non-surgical.

We considered only higher-level hospitals that received at least five referrals from a particular DH/MH as the standard RHs for the particular DH/MH. Referrals to non-surgical mono-specialist centres (e.g. mental health institutions) as well as lateral referrals (i.e. to hospitals of the same level) were excluded.

### Quantitative data analysis

Referral statistics and finance documents obtained from the DHs/MHs were entered, processed, and analysed using Microsoft Office Excel 2016. To explore a possible association between distances of referral trips and the numbers of referral trips, we made the scatterplots and estimated the Pearson product-moment correlation coefficients.

### Costing methods

For each hospital, the cost of fuel per round referral trip was first calculated by multiplying the volume of fuel consumed per trip by the prevailing cost of fuel per litre. In hospitals with no defined working estimates of fuel usage, we applied an estimate of 7 km/litre. Total annual costs of diesel used for referrals were obtained by multiplying the costs per referral by the total number of referrals per year.

Estimating the share of total ambulance maintenance costs attributable to referrals required certain assumptions, where ambulances were used for other activities besides patient referrals to higher centres. It was assumed that the shares of tear and wear attributable to referral trips were proportional to the shares of the total referral distances covered out of the entire distances covered by the vehicles over the period (as recorded in the logbooks), or the share of total fuel consumed for referrals out of the total fuel consumed by the vehicle in the year. These ratios were applied to the annual maintenance costs to obtain the costs of maintenance attributable to referrals. Average maintenance costs per trip were then obtained by dividing the total annual maintenance costs attributable to referrals by the total number of referral trips. Average allowance costs per trip were obtained by dividing the annual cost burden of allowances by the total number of trips.

Total annual costs of ambulance referrals were obtained by the summation of the annual costs of fuel, maintenance and allowances. Total costs of surgical ambulance referrals were obtained by multiplying the total annual costs of referrals by the surgical proportion of total patients referred. These total annual costs of surgical referrals were expressed relative to the total annual hospital expenditure in Tanzania, the total annual district health budget in Malawi, and the total annual hospital (government) grant in Zambia.

All cost data are presented in international dollars (I$) using the respective 2018 World Bank purchasing power parity (PPP) conversion factors.^1^

### Qualitative data analysis

Thematic content analysis was used. One researcher (MI) did a manual coding of interview notes based on the predetermined interview themes, while also looking out for other emerging (sub)themes. This was followed by axial coding, linking related concepts together. Initial results were shared with a broader group of researchers (HB, CP and LB) for a consensus on the thematic content.

### Ethical considerations

This study was approved by the National Institute for Medical Research – NIMR (approval no. NIMR/HQ/R.8a/Vol. IX/2600) and Kilimanjaro Christian Medical College – KCMC (approval no. CRERC 2026) in Tanzania; University of Malawi College of Medicine Research and Ethics Committee (COMREC) in Malawi; and University of Zambia Biomedical Research Ethics Committee (UNZABREC) and National Health Research Authority (NHRA) in Zambia. Informed consents were obtained verbally from interview respondents.

## Results

This section describes the findings of the study across the three countries in five subsections: referral webs, referral infrastructure, referral statistics, reported practices, and the financial costs of referrals. A selection of quotes from the respondents is included for illustration purposes. Some results are aggregated broadly across all the countries, and others presented at country levels for comparison purposes. Hospitals are anonymized only in the cases of sensitive discussions, while all respondents are totally anonymized.

### Referral webs

Table 3 presents the main RHS in the study settings in the three countries. A significant anomalous finding is the referral of all emergency cases from the northern region of Tanzania directly to the zonal hospital (KCMC), bypassing Mwenzi Regional Hospital. All the centres visited cited lack of confidence in the capacity of the regional hospital to provide timely care in emergency situations as the singular reason for this practice.

**Table 3:** Basic Referral Data Per Hospital

Country	District hospital	Main referral centre	Distance (one-way, in kilometres)	Number of referral trips undertaken in 12 months	Number of functional hospital ambulances + District ambulances
**Tanzania**	Meru	Mt Meru/KCMC	26^a^	624	2 + 5
	Oltrumet	Mt Meru	14	52	1 + 0 + ^b^
	Longido	Mt Meru	77	103	1 + 3
	Same	KCMC	110	384	1 + 4 + ^b^
	Hai	KCMC	27	425	2 + 0
	Huruma	KCMC	56	270	2 + 0
	Kilema	KCMC	40	416	1 + 0
**Malawi**	Mulanje	QECH	64	365	2 + 5
	Nsanje	QECH	220	180	4 + 3
	Mwanza	QECH	105	240	2 + 1^c^
**Zambia**	Namwala	Choma/LCH/UTH	278^a^	51	1 + 1
	Zimba	LCH	76	248	1 + 2
	Siavonga	UTH	200	22	1 + 1
	Nangoma	UTH	131	131	2 (1^c^)

^a^Weighted average distance; ^b^Mobile clinic; ^c^Temporarily grounded

*KCMC* Kilimanjaro Christian Medical Centre; *QECH* Queen Elizabeth Central Hospital; *LCH* Livingstone Central Hospital; *UTH* University Teaching Hospital

### Referral infrastructure

Table 3 also presents the number of ambulances available in each hospital and how many are available for intra-district patient transfers (from health centres to the DH). At all but one of the 14 centres, the managers reported inadequacies in numbers of ambulances, or poor functionality state, and inadequacies in patient support equipment in the ambulances. They also reported improvisations made to manage inadequacies in the numbers of ambulances. For instance, a particular centre reported fitting a regular utility vehicle with a siren to operate as an ambulance; even without the necessary patient support equipment.

### Referral trip distances and frequency of trips

**Fig. 1:**
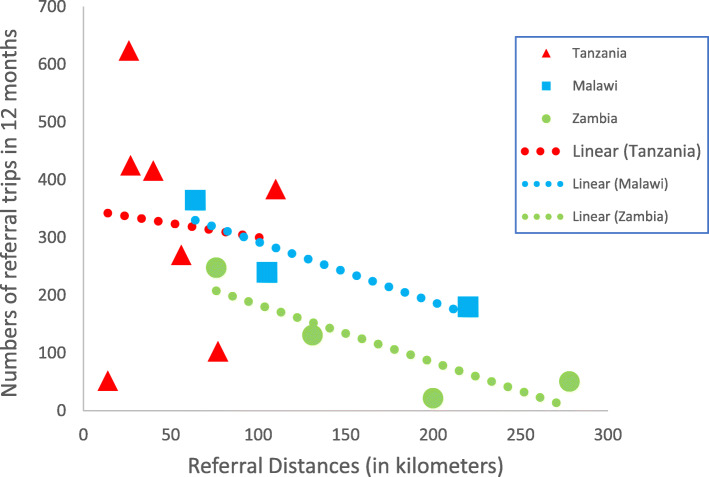
Referral Distances Correlated with Numbers of Referral Trips in 12 Months

The distances of the DHs to their respective RHs as well as the number of referral trips recorded in the year under review are also presented on Table 3. The distances vary widely and are generally shortest in Tanzania and farthest in Zambia. Figure 1 is a scatter plot of distances to referral center correlated with the numbers of referrals in a year. The correlation coefficients were negative but not statistically significant (α = 0.05; 95% confidence limit): -0.9, *P = 0.30* in Malawi; − 0.9, *P = 0.14* in Zambia and − 0.08, *P* = 0.9 in Tanzania.

### Referral practices

Referral practices vary across and within countries with regards to types of cases conveyed in the ambulance, documentation of referrals, ambulance management and maintenance, diesel use control, payment of allowances, and trip minimizing strategies.

#### Types of cases transported by ambulance

Cases conveyed by ambulance include emergency cases; patients arriving in critical states are offered initial support and then evacuated. Others are cases of sudden deterioration in otherwise stable inpatients. Referral shuttles also include inpatient cases that are “stable” but require specialist review or management. Elective cases are usually not transported by ambulance. However, it is common in Malawi for ambulances visiting the city for any other reason to provide “lifts” to Queen Elizabeth Central Hospital (QECH) for elective patients who usually hang around the DHs for such opportunities, and to convey home patients who have been discharged at QECH. Malawi DHs are mandated by the government to transport deceased patients from the RHs back to their respective communities.

#### Use of referral registers

Four DHs in Tanzania, two in Zambia, and none in Malawi were found to maintain comprehensive referral registers. The registers seen captured such data as the patient biodata (e.g. name, age, sex, and address), provisional diagnosis, name of accompanying personnel, name of the driver, and times of departure and return of the ambulance. None provided reasons for referral (e.g. lack of expertise, shortage of supplies or power failure) however.

#### Escorting personnel and allowances

All the hospitals reported “escorting” referred patients with appropriate health personnel although the thresholds differ. While some use escorts for every ambulance referral, others use escorts only when “extremely critical cases” are involved. Nurses are the most commonly assigned escorts. Depending on the nature and severity of the conditions, anaesthetists or clinicians (COs, MLs or MOs) may be involved.

At all the hospitals, respondents affirmed that the drivers and escorting personnel are entitled to allowances for travelling out of their primary stations of duty. Two government stipulated allowances were identified: one is daily subsistence allowance (DSA) or per diem (or night allowance), paid if the trip runs into or through the night. The rates vary based on the cadre, level, and destination status (urban versus rural). The reported rates were I$85 for a driver and average of I$90 for the nurses in Tanzania; I$38 and I$76 – I$114 respectively in Malawi; and I$133 and $167 respectively in Zambia. The other is the lunch allowance; paid if the trip was completed within the day time (or less than 8 h in Zambia). The reported rates were: about I$50 in Tanzania, I$15 in Malawi, and I$22 in Zambia.

Three hospitals in Tanzania, two in Malawi and three in Zambia reported paying the standard escort allowances. Two other hospitals in Tanzania reported paying only arbitrary lunch tokens of I$6 and I$17 to escorting nurses respectively. Some hospitals that reported not paying any allowances expressed more inclination to payment of night allowances should such arise. Across all the centres that reported paying any form of allowances, reports of irregularities such as delays, underpayment, or skipping of payments were common. Paucity of funds was the main reason cited for non-payment or irregularities in payment of escorting allowances.

Distance appears to be a strong factor influencing payment practices. The centres that pay no escorting allowances have an average referral distance of 60 km, compared to those that pay, which averaged 124 km from their referral hospital. A hospital manager gave insights into how their internal policy and the referral distance help them carry on without paying allowances:

*“Our policy is that nurses on duty go for referral trips when the need arises, therefore the trips are regarded as part of their routine duties, and the referral destination is close so there is never a situation where the worker will have to sleep over in the city”* (Respondent 49).

The manager however affirmed that non-payment of allowances affects staff motivation:

*“What happens is that when you are calling a staff to come and accompany a patient on a referral trip, he or she starts manufacturing all manner of reasons just to dodge the trip” (Respondent 49).*

#### Ambulance management

Two hospitals in Tanzania use their ambulances strictly for patient movements while the others occasionally use their ambulances for other activities, including conveying medical supplies, collection of blood from blood banks, and administrative activities. In Malawi, all three hospitals reported using the ambulance routinely for activities other than patient movement. In Zambia, only one hospital reported “sometimes” using the ambulance for medical supplies .

Across all three countries, ambulance maintenance was reported to be erratic: no hospital reported consistently servicing the vehicles after every 5000 km mileage, which was the reported standard. Delays in repair following vehicle breakdown were reported in all countries, with inadequacies in funding being cited as the reason for the delays. An accountant gave insights into this situation;

*“We are supposed to do routine maintenance four times a year ideally, that is after every 5000km, but now we do it after 10,000km [* … because of paucity of funds*]”* (Respondent 50).

A unique strategy was reported at Nsanje DH in Malawi. To cut down costs, all district health drivers were trained in basic routine vehicle servicing techniques, such as changing oil or essential vehicle parts. In this way, the District Health Officer (DHO) only has to provide the necessary parts while the drivers effect the replacements at no extra costs.

#### Use of non-ambulance vehicles for referrals

Two hospitals in Tanzania and two in Malawi reported occasionally using other non-ambulance vehicles to convey patients. Inadequacies in ambulances was the major reason for use of non-ambulance vehicles:

*“We had only one ambulance for long. Just last week we received a new ambulance. The church arranged some individuals to donate the ambulance. It was a big challenge having only one*. *We used other vehicles to convey emergency cases [...*when the ambulance was not on ground*]. When we do this we receive a permission from the police to exceed the speed limits. These are hospital vehicles but not ambulances. Rarely, in the case of multiple accidents we can use taxis to ferry plenty of patients.”* (Respondent 20).

#### Fuel management

All the hospitals studied were found to keep and use vehicle log books, although with varying degrees of strictness. In general, trips, purposes, destinations and distances are registered for each trip. Before fuel is approved for a particular ambulance, the current mileage reading is tallied against the mileage reading at the time of the last fuel supply to ascertain the distances covered since that point. A dedicated transport officer or any other delegated senior member of the administrative team is responsible for these checks. Dispensing of fuel to a vehicle (from the hospital stock) at the gas station in most cases is only on presentation of fuel “vouchers” signed by the appropriate district health or hospital manager.

Our findings indicate that the robustness and consistency of these systems indeed has a huge impact on the annual bill of the hospital. A particular hospital reported almost a 50% drop in the monthly fuel costs within one year of changes in management team.

*“As at 2 years ago, the hospital spent about 10M/month on just referrals alone. Since April last year, with better communication* [teleregulation of referrals from health centres*], new DHO, the change of the transport officer, and better scrutiny of the logbooks, the hospital now spends about 5.5 to 6M / month on referrals. These figures are for the entire district.”* (Respondent 29).

Again, a novel approach was recorded at Nsanje DH in Malawi where all the vehicles were fitted with GPS trackers. Hence, the hospital management could monitor more accurately the movements of all the vehicles in the fleet.

#### Trip minimizing strategies

DHs employ two main strategies to reduce trips and therefore cut down referral costs. The first is through transporting multiple patients. All the hospitals in Zambia and Malawi and three in Tanzania reported combining patients in single trips to save trip costs. Patients are commonly combined when two or more critical cases require immediate evacuation or in situations of multiple casualties due to road traffic accidents. A more cost-driven variant however is the practice of delaying and accumulating relatively stable patients who require specialist management in the wards, to accompany critical cases when they arise.

*“We combine the patients to reduce the costs of fuel. For instance we can combine an adult and a small child because the bed size is small. If they are stable enough to sit, two adults or even three can be transferred together. Combination of patients is frequent.*” (Respondent 20).

The second strategy is trip synchronization. In all the centres where ambulances are used routinely for non-referral activities, such other activities are synchronized with patient referral trips. For instance, an ambulance trip to a central hospital might visit the central medical stores in the same city as the central hospital to collect supplies before returning to the district. Stable patients are also delayed in the wards for evacuation to the central hospital on scheduled blood bank visit days when the ambulance would visit the city for blood collection.

#### Cost recovery

In Tanzania, the two missionary hospitals in the sample - Kilema and Huruma - reported charging fees of I$50 and I$33 respectively for referral services, except where patients are statutorily exempted from user fees. In situations where patients are combined, the charges are split. Across all the rest of the study hospitals, patients are conveyed free of charges.

#### Funding and budget ceilings

The main sources of funding for referrals are government operational grants (across all the hospitals) and hospital own funds (in Tanzania especially). All the hospitals capture referral costs in their annual budgets, either explicitly or subsumed under the broader item lines: fuel, vehicle/equipment maintenance, and allowances. Especially in Malawi and Zambia, different NGOs support referral services by funding the fuel, vehicle maintenance or allowances.

Across all the countries, there are government ceilings on allocations of government operational grants to fuel, allowances, and transport, at least in broad terms. However, compliance to these regulations is generally poor, and considered unrealistic by the managers.

*“Yes, there is a ceiling on expenditure on referrals. But this is the issue, I exceeded the fuel ceiling a long time ago. So tell me, are you saying if I have a patient to refer now I will tell him/her ‘sorry, I have exceeded the fuel ceiling’?”* (Respondent 60).

### Costs of referrals

**Fig. 2:**
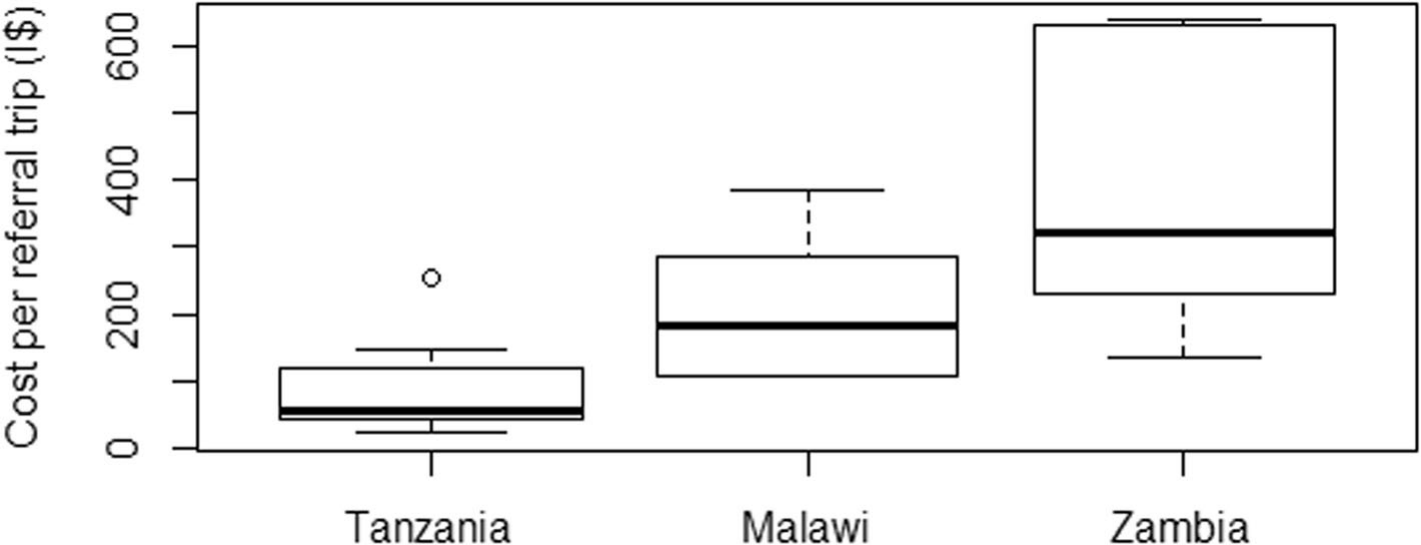
Boxplots of Unit Costs of Referrals in Tanzania, Malawi and Zambia

Table 4 shows the costs of all ambulance referrals and of those that involve surgical cases across the study centres, while Fig. 2 presents the boxplots of costs of single trips across the three countries. Unit costs of referrals varied widely across the countries and even within the same country. Expectedly, distance to the referral centre was the major factor determining the unit costs. In Tanzania, it ranged from around I$19 at Meru DH to I$208 at Huruma MH. In Malawi, the costs were I$91, I$153 and I$320 for Mulanje, Mwanza and Nsanje DHs respectively. In Zambia costs ranged from I$116 at Zimba MH with the shortest travel distance to I$538 at Namwala DH with the farthest travel distance.

**Table 4:** Costs of Ambulance Referrals Per Hospital

Country	DH	Cost per trip (I$)	Total annual costs of all referrals (I$)	Disaggregation into proportions of cost components (fuel: allowances: maintenance)	Surgical proportion of all referrals (%)	Annual costs of surgical referrals (I$)	Costs of surgical referrals expressed relative to total annual operational expenditure (%)
**Tanzania**	**Meru**	19	11,940	92: 0: 8	53^a^	6328	1
	**Oltrumet**	41	2128	28: 16: 56	53^a^	1128	0.52
	**Longido**	76	7873	86: 0: 14	54	4252	3
	**Same**	121	46,640	64: 32: 8	60	27,984	7
	**HAI**	29	12,281	78: 0: 22	44	5404	1
	**Huruma**	208	56,231	49: 29: 21	53^a^	29,802	3
	**Kilema**	46	5759	58: 20: 22	53^a^	10,221	0.6
**Malawi**	**Mulanje**	91	33,122	74: 1: 25	48	15,845	4% ^b^
	**Nsanje**	320	57,594	74: 16: 11	55	31,570	6% ^b^
	**Mwanza**	153	36,638	88: 0: 12	39	14,187	4% ^b^
**Zambia**	**Namwala**	538	27,448	42: 36: 22	74	20,312	17 ^c^
	**Zimba**	116	28,759	69: 0: 31	68	19,556	27^c^
	**Siavonga**	534	13,870	45: 13: 42	79	10,898	14^c^
	**Nangoma**	273	35,767	46: 47: 7	69	24,680	25^c^

^a^Obtained by taking the average of Longido, Same and Hai data; ^b^Expressed relative to planned expenditure (budget); ^c^Expressed relative to annual government grants; *DH* District Hospital

Total annual cost burdens of ambulance referrals also varied significantly, depending on the unit costs of trips and the volumes of referrals. The range in Tanzania was from I$2 k at Oltrumet DH to around I$56 k at Huruma DH. In Malawi, I$33 k, I$37 k, and I$58 k costs were incurred from ambulance referrals at Mulanje, Mwanza and Nsanje DHs, respectively. The lowest cost found in Zambia was $14 k at Siavonga DH while Nangoma MH spent the most at I$36 k.

A disaggregation of the costs into the components showed that fuel was the biggest cost driver across nearly all the centres, accounting for as much as 90% of the total costs in some cases. Oltrumet DH stands out with vehicle maintenance accounting for as much as 70% of the costs. This might be a reflection of the poor state of the vehicle which would warrant higher costs of maintenance.

The surgical proportions of all referrals were highest in Zambia, ranging from 69% to 79%; followed by 44% to 60% in Tanzania; and then 34% to 48% in Malawi. In absolute figures, the ranges are I$11 k – I$25 k; I$1 k - I$29 k; and I$14 k – I$32 k; respectively. These figures represent about 14–27% of government hospital operational grants in Zambia; 5% of annual DH budget in Malawi; and 0.6–7% of total DH operational expenditure in Tanzania.

## Discussion

Using a multi-methods, multicenter case study approach, this study is the first to quantify the financial burdens on DHs of surgical patient transportation by ambulance in SSA. Our study highlights the financial challenges of ambulance referrals for DHs and how clinicians and hospital managers navigate these. The total cost of ambulance use for patient transportation ranged from I$2 k – I$58 k per year. Between 34% and 79% of referrals were surgical, ranging between I$1 k and I$32 k per year. Resource constraints force hospitals to use their ambulances for other activities, or to use other non-ambulance vehicles to convey patients; or to delay, accumulate and combine patients in order to reduce trip frequencies and save costs.

The high proportions of surgical cases among the referrals indicate a big potential for savings if district level surgery is scaled up. Investing in numbers and skills of surgical personnel at the DHs, for instance, has been shown to reduce referrals substantially [32, 40–42]. Sani et al. (2009) demonstrated that a one year surgery training programme for general practitioners at DHs in Niger diminished emergency transfers to higher centres “drastically” [41]. Savings from decreased referrals could be channeled to other needs of the DHs. Although this study did not consider the “appropriateness” of the surgical referrals, a study conducted in Malawi by the same team found that one in three referrals received at QECH from the first level hospitals was “unnecessary”, as it should have been ideally managed at the lower level [17]. This represents a potential 35% savings in costs of surgical referrals through district level surgery scale-up.

Our earlier paper involving application of a systems dynamics tool (Group Model Building) revealed that scale-up requirements are complex and resource intensive [43]. National Surgical, Obstetric and Anaesthesia Plans (NSOAPs), recently adopted and currently being implemented in Tanzania [44] and Zambia [45] are widely considered as charting the right direction for surgical scale-up. However, unlike the Tanzania NSOAP, the Zambian plan did not capture explicitly the costs of developing and maintaining a robust and responsive ambulance transportation service. Further, although both NSOAPs propose to create distinct central level funding lines for district surgeries, this has not yet been implemented in either of the two countries. There is an overriding need to expedite actions in this regard as it is critical to the overall success of the NSOAPs.

Hospitals consider costs, at least implicitly, in making referral decisions, even though this study found no statistically significant association between referral trip distances and numbers of trips – a possible consequence of the small sample size in the study. Reflecting on the results in a workshop with a broader group of DH managers and other stakeholders in Lusaka, participants asserted that remote DHs are indeed relatively less inclined to undertake referral trips to central hospitals due mostly to costs reasons. A more robust study with a larger sample size would be appropriate to obtain finer insights into the distance-frequency dynamics.

Transport is critical for optimal patient care, and has been shown to make a difference between living and dying [38]; yet challenges of ambulance inadequacies remain widespread in SSA [46, 47]. In 2012, the 1000 vehicle national fleet (utilities and ambulances) of Zambia Ministry of Health had an average age of 7 years, and 40% of them were beyond economic repair or required substantial rehabilitation work [48]. Although patient outcome assessment was beyond the remit of this study, it nevertheless highlights some practices that are potentially detrimental to patient safety, or shift additional costs onto patients and their families, such as deliberate delays in transfer of patients perceived as “stable” and use of poor or non-ambulance vehicles.

An earlier study involving health centres in Zomba district of Malawi indicated that patients sometimes spent a night or two waiting for an ambulance to arrive [38], and the DH managers attributed this to inadequacies of ambulance, worsened by shortages in fuel supply. Use of non-ambulance vehicles due to lack of functional ambulances had been reported in Uganda [49] and Ghana [50]; and the difficulties faced by health workers in patient monitoring on account of such use of inappropriate vehicles for patient referrals, undermining patient safety, had also been documented [50].

From the insights provided in this study, several recommendations are derived towards the improvement of the referral infrastructure for cases that merit referral.

- First, provision of adequate numbers of ambulance vehicles to the hospitals is of utmost importance, with attention to the functionality and equipment of the vehicles to improve patient safety [51]. Ambulance procurement contracts should include maintenance plans with the vendors, while public institutions tasked with maintenance responsibilities such as Tanzania Electrical, Mechanical and Mechanical Services Agency (TEMESA) ought to be revamped to deliver effectively on their mandates.
- Second, as the referral infrastructures are currently overstretched, with few ambulances serving entire districts, there is a need to consider cheaper means of transport for intra-district referrals to free up vehicles for longer and more critical extra-district referrals. A previous study in Mangochi district of Malawi showed that motorcycle ambulances were more efficient and reduced intra-district referral delays by 2 to 4.5 h [25]. The purchasing cost and the annual operating cost of a motor ambulance were 19 times and 24 times higher than those of a motorcycle ambulance, respectively.
- Most importantly, there is a need for both generic national guidelines as well as specialty specific guidelines to regulate referral practices. As lack of confidence in the next level hospital was reported as a major reason why DHs bypass their hierarchically appropriate RHs, it is important to ensure availability of high quality services at all levels of the pyramid - to avoid delays in patient care where hospitals at different levels fail to provide appropriate standards of care [52].

There are indications that more could be achieved even with the limited available resources. Wastages and thefts are not uncommon in the public service in SSA [53] and health services are not immune from this [54]. A study of district health system efficiency in Malawi reported frequent leakages in hospital drugs and food stuff, manipulation of vehicle maintenance contracts, and staff enrichment from manipulation of fuel costs; all driven by erroneous perceptions of government resources as “free” resources, and/or due to poor staff remuneration [54]. Fuel and vehicle maintenance are the major drivers in the costs of ambulance referrals and warrant close monitoring. Our finding of an almost 50% fall in the fuel bill within one year, after the change of the DMO and the transport officer in one particular hospital, underscores what could be achieved with improvements in accountability and efficiency. Introduction of electronic vehicle tracking systems as seen at Nsanje district brings a new layer of security in fuel management, reducing the risk of logbook manipulation or odometer tampering. DHs should be encouraged to learn from each other to discover creative and more efficient approaches, such as the innovative strategies used at Nsanje DH. Above all, there is a real need for strengthening of monitoring frameworks across all the levels of the health system [54].

Lastly, there may be a need for reviews of national revenue allocation formulae for district health services in the three countries to accommodate geographical variations in patient referral costs. Facilities far flung from the RHs, e.g. Nsanje DH and Namwala DH, suffer disproportionately from the double impacts of their higher costs of diesel and staff allowances, not only because of the need to motivate workers to undertake such long trips, but also because referral trips are more frequently overnight. Although Tanzania and Malawi health resource allocation formulae include indices accounting for higher operational costs of intra-district healthcare delivery (including supplies distribution, immunization and supervision) in rural and sparsely populated areas [55, 56], extra costs of longer referral distances to the cities are not captured in any of the three countries. This effectively shortchanges the remote facilities.

### Limitations

First, the heterogeneity in how data were collected (i.e., the nature, sources, and the time frames covered) across the different hospitals makes cross-country and sometimes within-country comparisons difficult. Also, in some cases data on ambulance trips were not well documented, which required undue reliance on the recall or subjective estimates of hospital administrators. However, we made conscious efforts to triangulate every single data piece from as many points as possible.

Second, this study captured only the financial costs associated with referrals, leaving out depreciation and opportunity costs. For instance, there may be productivity losses with regards to in-hospital patient care while health workers accompany patients on referrals. Full economic costing of surgical ambulance referrals would have captured that.

## Conclusion

DHs in SSA face considerable pressures managing high volumes of surgical ambulance referrals in resource constrained environments. Hospital managers adopt certain coping strategies which are potentially compromising to patient safety and health outcomes. Asides the potential health benefits of district level surgery scale-up, our study provides economic argument for investments in strengthening the surgical capacity of the DHs, as savings from decreased referrals could be reinvested in referral systems, in particular ambulances. Optimum results will only be obtained from the referral system if district level interventions are complemented with appropriate surgical capacities at every level of the pyramid.

## Supplementary Information

**Additional file 1.** Interview respondents at the study hospitals.

**Additional file 2.** Interview guide.

## Data Availability

The datasets used and/or analysed during the current study are available from the corresponding author on reasonable request.

## Footnotes

1 PPP conversion factor, GDP (LCU per international $). 2020;https://data.worldbank.org/indicator/PA.NUS.PPP?view=map. Accessed 15th May 2020.
