# Supplementary material for: Surgical ambulance referrals in sub-Saharan Africa – financial costs and coping strategies at district hospitals in Tanzania, Malawi and Zambia
Source: BMC Health Serv Res. 2021 Jul 23;21:728. doi: 10.1186/s12913-021-06709-5 (PMC8299644; doi:10.1186/s12913-021-06709-5)
Supplement: Supplementary file 1 — Additional file 1. Interview respondents at the study hospitals. [file 12913_2021_6709_MOESM1_ESM.docx]

| DH | DHO (or representative) | MOI | Administrator/Secretary | Matron | Accountant | Other senior staff (eg Procurement officer, HRO) | TO | OPD in-charge(s) | Driver | Senior clinician(s) | Total |
| --- | --- | --- | --- | --- | --- | --- | --- | --- | --- | --- | --- |
| **Tanzania** |  |  |  |  |  |  |  |  |  |  |  |
| Meru DH | no | yes | no | yes | no | no | yes | Yes | no | no | 4 |
| Oltrumet | no | yes | no | no | no | no | no | no | no | Yes (2) | 3 |
| Longido | no | yes | no | no | yes | no | no | no | yes | no | 3 |
| Same | no | yes | yes | no | yes | no | no | no | no | yes | 4 |
| Hai | no | yes | yes | yes | yes | no | no | no | no | yes | 5 |
| Huruma | no | yes | no | yes | yes | no | no | no | no | yes | 4 |
| Kilema | no | yes | yes | no | yes | yes | no | no | no | yes | 5 |
|  |  |  |  |  |  |  |  |  |  |  |  |
|  |  |  |  |  |  |  |  |  |  |  |  |
| Subtotal |  |  |  |  |  |  |  |  |  |  | **28** |
|  |  |  |  |  |  |  |  |  |  |  |  |
| **Malawi** |  |  |  |  |  |  |  |  |  |  |  |
| Mulanje | yes | yes | yes | no | yes | no | yes | no | no | yes | 5 |
| Nsanje | yes | yes | yes | yes | yes | no | no | no | no | Yes | 7 |
| Mwanza | no | yes | yes | yes | yes | no | yes | no | no | no | 5 |
| Subtotal |  |  |  |  |  |  |  |  |  |  | **17** |
|  |  |  |  |  |  |  |  |  |  |  |  |
| **Zambia** |  |  |  |  |  |  |  |  |  |  |  |
| Namwala | no | yes | no | no | yes | no | no | no | no | no | 2 |
| Zimba | yes | no | yes | yes | yes | no | no | no | no | no | 4 |
| Siavonga | yes | yes | no | yes | yes | no | no | no | no | no | 5 |
| Nangoma | yes | yes | yes | no | yes | no | no | no | no | no | 4 |
| Subtotal |  |  |  |  |  |  |  |  |  |  | **15** |
|  |  |  |  |  |  |  |  |  |  |  |  |
| Grand Total |  |  |  |  |  |  |  |  |  |  | 60 |
|  |  |  |  |  |  |  |  |  |  |  |  |

DH = District Hospital; MOI = Medical Officer In-charge; TO = Transport Officer; OPD = Out-Patient Department; HR = Human Resource Officer. Staff members who had more than one portfolio were counted only in one category
