# Supplementary material for: Surgical ambulance referrals in sub-Saharan Africa – financial costs and coping strategies at district hospitals in Tanzania, Malawi and Zambia
Source: BMC Health Serv Res. 2021 Jul 23;21:728. doi: 10.1186/s12913-021-06709-5 (PMC8299644; doi:10.1186/s12913-021-06709-5)
Supplement: Supplementary file 2 — Additional file 2. Interview guide. [file 12913_2021_6709_MOESM2_ESM.docx]

**Interview Guide**

**SYSTEM OF REFERRALS *(MD)***

- What is the system or network of referral in this hospital
- Do you have a one way or a two-way referral system?
- Could you list all the facilities where you refer your patients?
- What are the estimated distances of these referral centres from this facility?
- How many vehicles do you have in this hospital?
- How many are ambulances and how many are for administrative purposes?
- How many of the ambulances are functional as we speak? If anyone is broken down, when did it break down?
- Do you at anytime use any of the ambulances for reasons other than patient transfer?
- Do you use any other vehicles (asides ambulances) to convey patients?
- If yes, which vehicles? in which circumstances?
- For instance, do you also use administrative vehicles or private vehicles of staff members to convey patients to the receiving points?
- Do patients pay when they are conveyed to referral centers with a hospital vehicle? If yes, who pays? what is the rate?
- Are there situations where the ambulance has to take two patients at the same time?
- Do you also convey non-emergency or elective cases to the receiving facilities?
- When referring patients by ambulance is there always medical personnel to accompany a patient to a referral facility?
- Are there situations where referral centers use ambulances to convey patients back to you?

**SURGICAL REFERRAL STATISTICS *(MD)***

- Do you ever have surgical referrals from here?
- Do you have records of all your referrals in the last one year? (Can we access this?)

*** If records are available, check for 2017/2018, 2018/2019 FYs*

- Could you estimate the total number of cases you referred to other centres in the last one year? (**OR** Could you advise us on how best to obtain a reasonable estimate?:ward registers, hmis data, opd records, transport)
- How much of these are surgical cases?
  - %ages going to the various referral centres
  - %age electives and emergencies
  - %age being ferried by hospital ambulance
  - %age conveyed by hospital admin vehicles (if any)
  - %age conveyed by private arrangements
- List of various surgical conditions referred and their respective occurrences (proportions)
- Could you list the reasons for referral of surgical patients and their respective occurrences?

**COSTS (MD/Transport Officer)**

- What are the costs are associated with referrals?
- List of all costs items
- Volume per trip
- Unit Cost
- Are drivers and/or nurses paid any allowances for transporting patients? If yes, what is the rate?

**FUEL *(MD/Accountant/Transport Officer/Driver)***

- List of all the vehicles available, years of acquisition, and functions
- How do you fuel your vehicles?
- Estimates of the fuel consumption rates for the different vehicles in terms of per km and per week
- How do you source fuel for your vehicles?
- Is it sourced centrally or is it decentralized?
- Who bears the cost?
- What is the system of allocation or distribution of fuel to the various vehicles?
- How much fuel do you use in a month for each ambulance/vehicle?
- How much do you spend in a month for fueling each ambulance/vehicle?
- Do you have an estimate of how much fuel spent in each referral or trip?
- Do you have a logbook of your movements? Can we access this?
- Can we have a record of your fuel supply and consumption for the last one year?

**INDIRECT COSTS (*MD/Accountant/Transport Officer/Driver*)**

- Scheduled/Routine maintenance (tyres, batteries, servicing etc)
  - Costs per interval
  - Frequency
  - Estimated cost for the year
- Unscheduled maintenance (Breakdown repair)
  - Frequency
  - Estimated cost per year
